# Extracellular Endothelial Cell-Derived Vesicles: Emerging Role in Cardiac and Vascular Remodeling in Heart Failure

**DOI:** 10.3389/fcvm.2020.00047

**Published:** 2020-04-15

**Authors:** Alexander E. Berezin, Alexander A. Berezin

**Affiliations:** ^1^Internal Medicine Department, State Medical University, Ministry of Health of Ukraine, Zaporozhye, Ukraine; ^2^Internal Medicine Department, Medical Academy of Post-graduate Education, Ministry of Health of Ukraine, Zaporozhye, Ukraine

**Keywords:** extracellular vesicles, cardiac and vascular remodeling, heart failure, epigenetics, co-morbidities

## Abstract

Extracellular vesicles play a pivotal role in numerous physiological (immune response, cell-to-cell cooperation, angiogenesis) and pathological (reparation, inflammation, thrombosis/coagulation, atherosclerosis, endothelial dysfunction) processes. The development of heart failure is strongly associated with endothelial dysfunction, microvascular inflammation, alteration in tissue repair, and cardiac and vascular remodeling. It has been postulated that activated endothelial cell-derived vesicles are not just transfer forms of several active molecules (such as regulatory peptides, coagulation factors, growth factors, active molecules, hormones that are embedded onto angiogenesis, tissue reparation, proliferation, and even prevention from ischemia/hypoxia), but are instead involved in direct myocardial and vascular damage due to regulation of epigenetic responses of the tissue. These responses are controlled by several factors, such as micro-RNAs, that are transferred inside extracellular vesicles from mother cells to acceptor cells and are transductors of epigenetic signals. Finally, it is not a uniform opinion whether different phenotypes of heart failure are the result of altered cardiac and vascular reparation due to certain epigenetic responses, which are yielded by co-morbidities, such as diabetes mellitus and obesity. The aim of the review is to summarize knowledge regarding the role of various types of extracellular endothelial cell-derived vesicles in the regulation of cardiac and vascular remodeling in heart failure.

## Introduction

Heart failure (HF) is a complex condition which is often accompanied by co-morbidities and a high prevalence in the general population, and is a final stage of various cardiovascular (CV) diseases (1). Despite sufficient improvements in diagnosis, prevention, and treatment of HF, new incidences of HF with reduced ejection fraction (HFrEF) and mid-range ejection fraction (HFmrEF) continue to occur due to a poor prognosis and need for mechanical support devices and heart transplantation (2, 3). The nature of the evolution of HF is tightly associated with substantial structural cardiac and vascular remodeling that is controlled by both genetic and epigenetic factors (4). Previous preclinical and clinical studies have revealed that epigenetic mechanisms, including chromatin modifications and non-coding RNAs, have emerged as molecular transducers of age, etiology triggers and co-existing metabolic factors, environmental stimuli, and inflammatory and neurohumoral regulatory molecules to control gene expression (5, 6). In fact, pre- and post-ischemic conditioning, post-ischemic injury, oxidative stress and hypertrophic remodeling, endothelial dysfunction, accelerating atherosclerosis, plaque rapture, microvascular inflammation and occlusion, thrombosis and sub-intimal lipids' modification, extracellular matrix accumulation and cardiac/vessel fibrosis are the processes which may be potentially regulated by underlying altered chromatin modifications and non-coding RNAs dyshomeostasis in HF (7–9).

Extracellular vesicles (EVs) are a wide range of particles that are released from the most viable cells and transfer active molecules, such as hormones, regulatory peptides, growth factors, and chromatin, and play a pivotal role in cell-to-cell cooperation, immunity, inflammation, apoptosis, and repairs (10). Developing HF adds to EVs' formation from the numerous types of cells including cardiac myocytes, fibroblasts, mononuclear cells, platelets, endothelial cell, progenitor cells, and even stem cells (11). Endothelial cell-derived EVs are a secretome of the progenitor and mature endothelial cells and are involved in functional and structural repairs of myocardium, endothelium, and vascular vasculature (12). Therefore, chromatin materials are able to be transferred as a cargo with EVs from cell to cell due to cell activation or apoptosis and thereby influence target cells acting as epigenetic factors (13). Finally, the epigenetic changes may influence many intercellular communication signaling systems, including the nitric oxide, angiotensin, and endothelin-1 signaling systems, which are embedded onto pathogenesis of cardiac and vascular remodeling (14, 15). The aim of the review is to summarize knowledge regarding the role of various types of extracellular endothelial cell-derived vesicles in the regulation of cardiac and vascular remodeling in HF.

## Extracellular Vesicles: Definition and Nomenclature

Previously secreted membrane-enclosed particles, which are collectively called extracellular vesicles (EVs), include exosomes, ectosomes, microvesicles, small size microvesicles, microparticles, nano particles, apoptotic bodies, and other EVs. Some of them (ectosomes and microparticles) were not determined as distinct from each other, and several classification approaches (sedimentation speed-derived criteria, immune phenotype, origin, mechanism of release, and size) were applied to EVs' subsets to qualify them in some classes. According to the Executive Committee of the International Society for Extracellular Vesicles, EVs are defined as mixture particles ranging from 30 to 2,000 nm in diameter, which are released by various types of viable cells in several different mechanisms (blebbing and budding of endosomal or plasma membranes) and they include exosomes, microvesicles, and apoptotic bodies (16). Table 1 reports nomenclature and basic characteristics of several subtypes of EVs.

**Table 1:** Nomenclature and basic characteristics of several subtypes of EVs.

**Characteristics of EVs**	**Subpopulations of EVs**		
	**Exosomes**	**Micro vesicles (ectosomes)**	**Apoptotic bodies**
Diameter, nm	40–100	100–1,000	50–2,000
Origin	Endocytic membrane	Cell membrane	Apoptotic cells
Mechanism of delivery	Ceramide-dependent, tetraspanin-dependent, and ESCRT-dependent exocytosis of multi vesicular bodies	Ca2+ depending phospholipid redistribution and Rho-kinase-mediated myosin light chain phosphorylation, facilitating budding, and blebbing	Thin membrane protrusion and blebbing of the apoptotic cells' surface
Phosphatidylserine composition	Low	High	High
Complexity/granularity	High	High	Low
Components	Cytoplasmic and membrane molecules, proteins and lipids, tetraspanin's receptors	Adhesive molecules (ICAMs, PECAM-1, MCAM), membrane regulatory proteins (Rab), lipids (SpL, PL, LPS, LPS), and receptors (tetraspanin's receptors, LAIR-1, EGFR), enzymes (Rab GTPase, ERK, MLCK, TPI-1, HMGCL), immune system proteins (CD14, CD276, MiC-11), apoAII, SOD, β-actin, α-actin-4, HSP90AB1, cytochrome complex, SCP-2	Mitochondria, MHC II molecules, ICAM-3, phosphatidylserine, sialylated and glycosylated ligands
Nuclear fractions	mRNA and microRNA (miRNA), other non-coding RNAs	Non-coding RNAs	Non-coding RNAs
Specific surface markers	Tetraspanins (CD9, CD63, CD 81), ESCRT machinery proteins (Alix, tumor susceptibility gene 10), flotillin-1	CD40, Phosphatidylserine, integrins, selectins, ESCRT machinery proteins (Alix, Vps4)	Annexin V+, phosphatidylserine, caspase 3, histones
Key functional role	Cell-to-cell communication, cargo	Cell-to-cell communication, cargo	Cell-to-cell communication, cell clearance

*SOD, superoxide dismutase; HSP, heat shock protein; SCP-2, Sterol Carrier Protein 2; TPI-1, Triosephosphate Isomerase 1; HMGCL, 3-Hydroxy-3-Methylglutaryl-CoA Lyase; ESCRT, endosomal sorting complexes required for transport; ERK, a prototypic mitogen-activated protein kinase*.

### Exosomes

Exosomes are derivates of the endocytic membrane that have an average diameter of 40–100 nm and are released from several types of cells after exocytosis and the shaping of multivesicular bodies (MVBs) (17, 18). MVBs move along intracellular tubules, fuse with plasmatic membranes, and release exosomes onto extracellular space. Exosomes have various cellular components including cytoplasmic and membrane molecules, proteins, hormones (aldosterone), growth factors (vascular endothelial growth factor, transforming growth factor), cytokines (interleukin [IL]-1β, IL-6, IL-8), and lipids, and may also contain fragments of chromatin, such as non-coding RNAs and several inactive forms of micro-RNAs (17, 18). There are a common set of membranes and cytosolic proteins, which are embedded onto exosomes originated from distinct types of cells (19). The specific surface markers that ensure recognition of the exosomes are tetraspanins (CD9, CD63, CD 81), ESCRT (endosomal sorting complexes required for transport), machinery proteins (Alix, tumor susceptibility gene 10), and flotillin-1 (20).

### Microvesicles

Microvesicles (equally known as microparticles or ectosomes) typically have a range from 100 to 1,000 nm in diameter and are shaped as a result of budding of the cell membrane (21). Microvesicles are heavily enriched in phospholipids, such as phosphatidylserine and phosphatidylcholine, and numerous membrane-depended structures (receptors, CD markers) originated from the parent cells (22). Proteomics and lipidomics arrangement of microvesicles is extremely variable and includes membrane regulatory (Rab, Sterol Carrier Protein 2) and structure (β-actin, α-actin-4) proteins, heat shock protein HSP90AB1, adhesive molecules (ICAMs, PECAM-1, MCAM), lipids (SpL, PL, LPS, LPS) and receptors (tetraspanin's receptors, LAIR-1, EGFR), enzymes (superoxide dismutase, Rab GTPase, cytochrome complex, Akt/ ERK, triosephosphate isomerase−1, 3-Hydroxy-3-Methylglutaryl-CoA Lyase), immune system proteins (CD14, CD276, MiC-11), and apo-lipoproteins (apo-A-II) (23–25). Therefore, microvesicles may yield several non-coding RNAs and chromatin fragments coupled with the complexity of the other components (26).

### Apoptotic Cell-Derived Extracellular Vesicles

**Figure 1:**
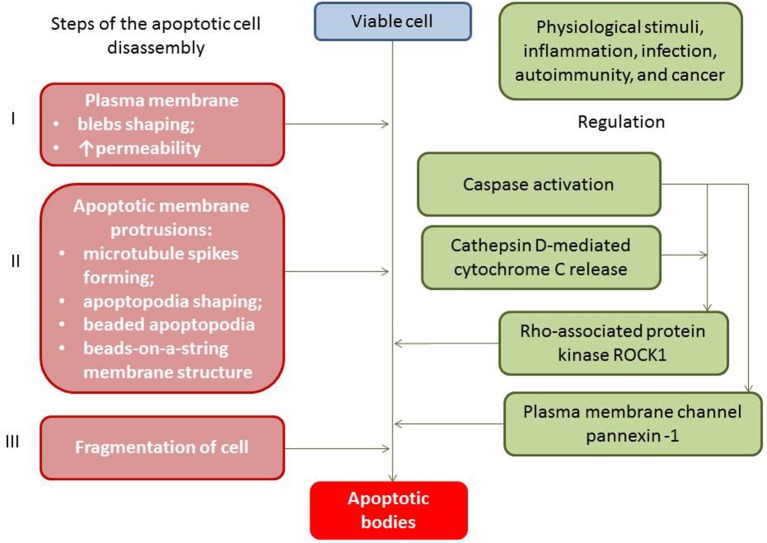
Apoptotic bodies generation and regulation.

Apoptotic cell-derived EVs include two types of apoptotic bodies: large membrane-bound vesicles (large apoptotic bodies [ABs] with diameter ≥1,000 nm) and small apoptotic microvesicles (small ABs with diameter <1000 nm) (27). Apoptotic bodies (ABs) are particles generally larger in size in comparison to both exosomes and microvesicles, while ABs have a variable diameter that fluctuates around 1,000 nm (from 1,000 to 2000 nm) (28). Both subpopulations of ABs result in blebbing of the surface of the apoptotic cells and contain regulatory specific proteins, numerous cell organelles, and chromatin fractions, like non-coding nucleus or nucleolus RNAs (29). The process of ABs' generation is precisely controlled by several distinct morphological steps (i.e., membrane permeability and blebs, membrane protrusion, and cell fragmentation), which are consequently regulated by several molecular factors including the Rho-associated protein kinase and the plasma membrane channel pannexin-1 (Figure 1).

ABs contain mitochondria, MHC II molecules, ICAM-3, phosphatidylserine, sialylated and glycosylated ligands, fragments of chromatin granules, DNAs, and non-coding RNAs. It has to be noted that the packaging of chromatin content (DNAs and non-coding RNAs) into the structure of the ABs is regulated during apoptosis and there are ABs that have no fragments of chromatin or remarkably low amounts of DNAs (30). ABs are classified depending on their origin from the mother cells including antigen-presenting cells, mononuclears, endothelial cells, fibroblasts, cardiac myocytes, and epithelial cells (31). The clearance of ABs has been ensured by phagocytes (32). To accurately differentiate ABs from other particles, such as cells and debris, there are several specific surface markers, such as Annexin V+/ phosphatidylserine (33).

## Biological Function and Pathological Role of Extracellular Vesicles

**Figure 2:**
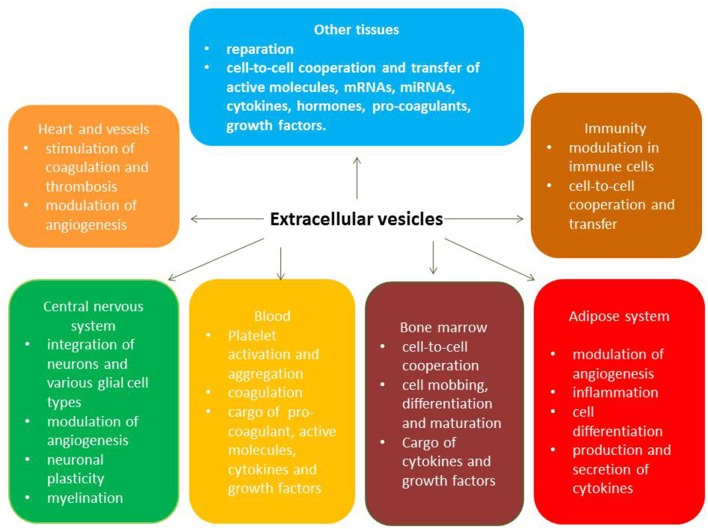
Pleotropic effects of circulating extracellular vesicles.

The key biological functions of EVs typically originate from various viable cells that use cell-to-cell communication and transfer materials called secretome. Acting as cargo for numerous molecules (Heat shock proteins [HSP-90, HSP-70], ILs, tumor necrosis factor-alpha, active molecules, enzymes, peptides, growth factors), EVs are recognized by target cells through specific antigens' presentation, bind to target cells, fuse with them, and abundantly supply the packaged materials to the cells. Therefore, exosomes and microvesicles naturally have a wide range of pleiotropic biological functions including immune response, antigen presentation, and the transfer of RNA and DNAs (28, 34). The full spectrum of pleiotropic effects of circulating EVs is reported Figure 2.

Recent studies have revealed that EVs may contain inactive forms of non-coding RNAs, which can be properly transferred to another cell and be functional in that new microenvironment (35, 36). Although 585 microRNAs were found to be up-regulated in HF patients, and 4,623 microRNAs were found to be down-regulated, most of them are circulating extracellular microRNAs, but a much smaller portion is transported using EVs (10, 26, 35). Indeed, under ischemia/hypoxic conditions, STEMI, HF, the up-regulated myocardial expression of pro-fibrotic (transforming growth factor [TGF]-β, growth differentiation factor 11 [GDF-11] and Rho-associated coiled-coil containing kinase-2 [ROCK-2]), and pro-inflammatory (inducible NO synthase, nuclear factor-kB, IL-2, IL-8, CCL5, STAT1, VEGF, TNF-alpha) genes and down-regulated gene expression of the matrix metalloproteinases (MMP-1, MMP-3, MMP-9) and their tissue inhibitors were found (35–37). In fact, EVs-related transfer microRNAs that have demonstrated abilities to up- (microRNA-210. microRNA-132) and down- (microRNA-17-3p, microRNA-222) regulate these genes through several intracellular signaling mechanisms (extracellular signal–regulated kinases 1/2 [ERK1/2], heat shock protein 27 (HSP27) signaling).

There is strong evidence that hypoxia and ischemia are triggers for mononuclear-depending production of pro-inflammatory cytokines including IL-2 and TNF-alpha, while supply of these cytokines to the target cells mediates through the package into EVs (37). On the contrary, HSPs, growth factors, non-coding RNAs, and active molecules, which are transferred by EVs, are involved in the regulation of reparative response, immunity reaction, and mediating cytoprotection (38, 39). However, a wide spectrum of biological active molecules that are transported by EVs from mother cells to the target cells yielded the ability to regulate endogenous repair system activity including proliferation, differentiation and mobbing of progenitor cells, and angiogenesis (40, 41). Through appropriate receptor-ligand (integrin αvβ3, CD40 ligand, neuregulin 1, VE-cadherin and beta-catenin) interactions and content cargo, EVs are able to regulate intracellular signaling pathways ensuring the activation of endothelial cells and the attraction and internalization of various circulating blood cells (platelets, mononuclears, macrophages, lymphocytes) to the endothelial cell surface (41). Moreover, vascular growth, restoring vascular integrity and function, and the recruitment of inflammatory cells may be directly related to up-regulated expression of the neuregulin 1 in the endothelial cells in results of EV-depended stimulation, because circulating EVs can be a source of variety of pro-angiogenic mRNAs including mRNA neuregulin 1 (42). Additionally, EVs may naturally induce a cytoskeleton-junction response of endothelial cells that is properly characterized by myosin light chain phosphorylation, contractile fiber reorganization, VE-cadherin phosphorylation, and adherent junction dissociation. This process is a key mechanism in the permeability of the vascular wall, release of neutrophil extracellular traps containing citrullinated histones and myeloperoxidase, and development of senescence and accelerating atherosclerosis (43–45). Proteome of EVs contains pro-coagulant components, such as tissue factor and phospholipids, which play a pivotal role in coagulation and the triggering of vasoocclusions in several diseases (46, 47).

## Extracellular Vesicles and Nature Evolution of Heart Failure

There is evidence that various cells in the failing heart and vasculature including cardiomyocyte progenitor cells, cardiac fibroblasts, circulating blood cells, and mature and progenitor endothelial cells, are largely mediated by the paracrine release of EVs conveying the reparative potency. Although transcriptomics and proteinomics of these cells have been widely investigated, the role of paracrine factors, such as EVs, in the regulation of cardiac and vascular remodeling in HF has not been fully understood.

### Cardiomyocyte Progenitor Cell-Derived EVs

Previously, cardiomyocyte progenitor cell (CPC)-derived EVs have shown beneficial effects on cardiac function and remodeling throughout the enhancement of the differentiation of cardiac progenitor cells into cardiac cells (48). CPC-derived EVs strongly inhibit lymphocyte and monocyte proliferation, suppressed inflammation, and prevented extracellular matrix accumulation (49). Indeed, CPC-derived EVs have significantly lowered the levels of pro-inflammatory cytokines, such as IgG1, IgG4, IgM, IL-1α, IL-2, IL-6, and TNF-alpha, among end-stage HF patients (48, 50). Therefore, CPC-derived EVs have reduced the number of pro-inflammatory Ly6Chigh monocytes, M1 macrophages, and suppressed NK cell degranulation in myocardium, while increasing the number of anti-inflammatory M2 macrophages (50). In fact, corresponding changes in the transcriptomic signature of the cardiac myocytes, CPC-derived EVs have demonstrated an ability to decrease tissue stiffness and BNP release and exhibited beneficial effects with regard to post-STEMI remodeling (49). Additionally, CPC-derived EVs contain a distinct repertoire of biologically active miRNAs, such as microRNA-373 and microRNA-21, that have strongly yielded anti-fibrotic effects and ameliorated fibrosis in the infarcted area targeting key pro-fibrogenic genes, i.e., TGF-β, GDF-11, and ROCK-2 (51, 52). Interestingly, EVs significantly inhibited microRNA-21 degradation and thereby mediate the anti-apoptotic effect in cardiac myocytes and endothelial cells (53). It has been demonstrated that the paracrine inhibitory impact of CPC on both cardiac fibroblast activation and collagen synthesis continues through cross-talk between cardiac fibroblasts and CPC-derived EVs (54). Thus, CPC-derived EVs ensure cardiac protection through paracrine output regarding cardiac myocytes that is attributable to decreased production of pro-healing cytokines and increased anti-inflammatory and anti-fibrotic microRNAs (55).

### Circulating Blood Cells-Derived EVs

Previous clinical studies have shown that there were no significant differences in the circulating number of EVs derived from platelets (CD41a+), neutrophils (CD66b+), erythrocytes (CD235a+), monocytes (CD14+), T lymphocytes (CD3+), and B lymphocytes (CD19+) between healthy volunteers and HF patients (56). In contrast, a decreased number of circulating endothelial cells (CD31+CD41a-) EVs was found in HF patients (57). However, the total number of EVs enriched phosphatidylserines was significantly increased in HF patients compared with healthy volunteers (56). In fact, an increased number of phosphatidylserines EVs derived from various cells, including platelets and erythrocytes, was associated with hypercoagulability of HF and mostly related to atrial fibrillation and reduced LVEF (58, 59). However, EVs derived from circulating blood cells other than endothelial cells are unlikely to play a significant role in the pathogenesis of HF, but several co-morbidities (diabetes, atrial fibrillation, chronic kidney disease, chronic obstructive pulmonary disease) may have a direct effect on EV releasing from blood cells and, thereby, exacerbate clinical evolution of the HF via pro-inflammatory and pro-coagulative potencies.

### Extracellular Endothelial Cell-Derived Vesicles

Extracellular endothelial cell-derived vesicles are released in both progenitor and mature endothelial cells after activation or apoptosis. The main triggers for EVs' synthesis and secretion vary depending on the presentation of various co-morbidities, the stage of HF evolution, medication use, as well as the implementation of mechanical support devices.

Innate molecular mechanisms of cardiac and vascular remodeling in HF has been investigated from several directions, such as myocardial hypertrophy and fibrosis, myocardial and microvascular inflammation, and myocardial mitochondrial dysfunction, as well as autophagy, apoptosis, and reparation. In fact, EVs play a pivotal role in various stages of the nature evolution of HF and mediate the pathological processes mentioned above (Table 2).

**Table 2:** EV-related pathways to regulate cardiac and vascular remodeling.

**Components of remodeling**	**Molecules transferred by EVs**	**Molecular mechanism/pathway**	**References**
Myocardial hypertrophy	G protein-coupled apelin receptor	Internalization through clathrin-mediated endocytic pathway	(60)
	long noncoding RNA Mhrt	Acetylation of myocardin with re-programming cardiac myocytes	(61)
	Micro-RNA-1,−155	Interaction with IGF-1, IGF-1 receptor and twinfilin-1	(62, 63)
Myocardial fibrosis	MMP-2, MMP-6, MMP-9	Direct degradation of collagen matrix and attenuation of LV dilation	(64, 65)
	Thymosin β4, FAP-α	Disproportionally distribution and arrangement of type I collagen fibers	(65)
	Micro-RNA-18,−19,−21,−22,−29,−30, - 133	Interaction with IGF-1, IGF-1 receptor, and PI3K/Akt/MAPK- NF-κB signaling pathways	(66)
	Micro-RNA-21	Inhibition of the extracellular inhibitor of the Spry1	(67)
	Micro-RNA-29	Interaction with the genes encoding the ECM, such as collagen, fibrillin, and elastin	(68)
Myocardial and microvascular inflammation	TNF-α, IL-6, IL-10, IL-18, CRP, HIF-1-α, NF-κB, micro-RNA-125a,−125,−138,−146,−155a	Erk1/2 STAT, Akt/MAPK- NF-κB signaling pathway NLRP3 inflammasome-activated IL-1β and IL-18 pathway VEGF/Akt and Eph/Ephrin signaling	(69)
Mitochondrial dysfunction	ROS, SOD, angiotensin II	↓ mitochondrial ATP synthesis, ↑ ROS production, ↑ fatty acid oxidation	(70, 71)
Autophagy	ROS, chemokines, chaperones, HSP-90, micro-RNA-145	mTOR-dependent pathway, Beclinl-dependent pathway,	(72, 73)
Apoptosis	ROS, HIF-1-α	Capsase-3-depended pathway	(74)
Angiogenesis	VEGF, IGF-1, VEGF-microRNA, VE-catherine, micro-RNA-	VEGF/Erk1/2 STAT—and PI3K/Akt/MAPK- NF-κB signaling pathways	(75)
Reparation	Thymosin β4, FAP-α, VEGF, IGF-1, VEGF-microRNA, TGF-β	Wnt1/β-catenin-depending signaling, VEGF/Erk1/2 STAT pathway	(76–78)
	microRNA-124,−126-3p,−508-5p	PI3K/Akt/MAPK- NF-κB signaling pathways	(79)
Immune activation	Micro-RNA-146 a/b,−223	Interaction with antigen-presenting cells, mononuclears	(65, 80)

*Mhrt, myosin heavy chain-associated RNA transcript; IL, interleukin; LV, left ventricular, NF-kB, nuclear factor-κB; TNF, tumor necrosis factor; ROS, reactive oxide species; SOD, super oxide dismutase; mTOR, a serine/threonine protein kinase; HSP, heat shock proteins; VEGF, vascular endothelial growth factor; IGF-1, insulin-like growth factor-1; TGF-β, transforming growth factor β; HIF-1-α, hypoxia-inducible factor-1 α; FAP-α, Fibroblast activation protein α; Spry1, sprout regulated kinase 1; ECM, extracellular matrix*.

In fact, at early stages of nature evolution of HF, the circulating levels of EVs derived from activated endothelial cells were higher when compared with healthy volunteers, while the levels of apoptotic endothelial cell-derived EVs were similar in stage A HF patients and healthy volunteers (81, 82). Therefore, numerous metabolic risk factors, such as resistance to insulin, hyperglycemia, abdominal obesity, and hyperuricemia, are considered to be early triggers for the mobilization of endothelial progenitor cells from bone marrow and peripheral tissue. These factors can also influence the transformation of several cells, such as fibroblasts and smooth muscle cells of vasculature into cells with endothelial cells' phenotype (83–86). This process is under strong epigenetic control and circulating EVs originated from activated and apoptotic endothelial cells and their precursors are able to regulate the repair of tissues such as endothelium and vasculature myocardium through attraction of cells with high innate ability to post-natal transformation (87, 88). Finally, increased levels of extracellular activated endothelial cell-derived vesicles characterize a tendency in endogenous repair systems to restore the integrity and function of target organs including the endothelium, myocardium, kidney, and brain (89).

Previous clinical studies have shown that the number of circulating EVs produced by progenitor precursors of endothelial cells or mature endothelial cells declines depending on the severity of HF, and patients with HFrEF had significantly lowered levels of EVs when compared with patients with HFpEF (90–92). In contrast, the advance of HF was associated with a steady increase in the circulating levels of apoptotic endothelial cell-derived EVs and gradual development of deficiencies in the pool of activated endothelial cell-derived EVs (93). However, lowered number of circulating EVs originated from activated endothelial cells was determined to be a marker of endothelial dysfunction with possible discriminative value to all-cause mortality, cardiovascular mortality, a risk of acute HF and acute decompensated HF onset, and an admission due to HF (94). Some evidence suggests that the ratio between the number of EVs derived from activated and apoptotic endothelial cells may yield a pronouncedly higher predictive potency for clinical outcomes intimately related to HF than a simple amount of EVs originated from several cell subpopulations (95).

**Figure 3:**
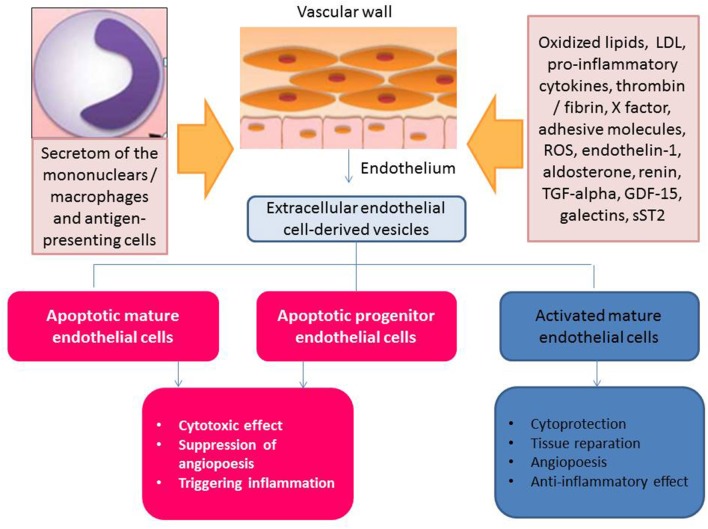
Apoptotic endothelial cell-derived and activated endothelial cell-derived extracellular vesicles: the role in HF pathogenesis.

**Figure 4:**
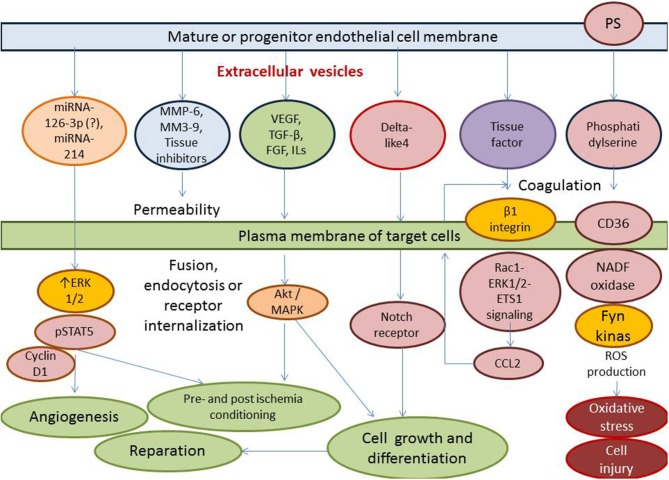
Molecular mechanisms ensuring the protective and deteriorating impact of endothelial cell-derived EVs on the target tissues (endothelium, vasculature, and myocardium). ROS, reactive oxide species; TGF, transforming growth factor; VEGF, vascular endothelial growth factor; MMP, matrix metalloproteinase; miRNA, micro ribonucleic acid; MAPK, mitogen-activated protein kinase; PS, phosphatidylserine; NADF, nicotinamide dinucleotide phosphate; CCL2, chemokine ligand−2; Rac1, Ras-related C3 botulinum toxin substrate 1; ERK1/2, extracellular signal-related kinase 1 and 2.

Thus, clinical data received from numerous investigators have indicated that the deficiency of the circulating activated endothelial cell-derived EVs and/or increased number of apoptotic endothelial cell-derived EVs might have a discriminative capability in HF with different phenotypes. This fact can be met with several difficulties, while the principal scheme regarding the role of activated and apoptotic endothelial cell-derived EVs in HF is reported in Figure 3. It has been suggested that organ protective effect is ensured by activated endothelial cell-derived EVs rather than apoptotic endothelial cell-derived EVs. Perhaps, proteinomics (β1 integrin, vascular endothelial growth factor, fibroblast growth factor-2, platelet-derived growth factor, enzymatic activity of matrix metalloproteinase [MMP]-2, MMP-6 and MMP-9), lipidomics (sphingosine-1-phosphate), oxidative stress components and enzymes (oxidized lipids, superoxide dismutase), non-coding RNA (micro-RNA [miRNA] 126-3p, mi-RNA-214, mi-RNA-125a, mi-RNA-150) profiles, and chromatin fragments are sufficiently distinguished in both subsets of EVs. There are several molecular mechanisms, which mediate the protective and deteriorating impact of endothelial cell-derived EVs on target tissues (Figure 4). In fact, endothelial cell-derived EVs are able to promote the protective effect that is associated with angiogenesis, tissue reparation, and pre- and post-conditioning due to VEGF/Erk 1/2 pSTAT- depending signaling pathway, whereas stimulation of Fyn kinases results in the internationalization of EV tissue factors with β-integrin, degradation of MMPs including neprilysin and C-reactive protein-embarked EVs provoke oxidative stress, cell injury, coagulation, and increase in vascular permeability, respectively (96, 97). Yet, EVs enriched Nox2-NADPH oxidase micro-RNA and insulin growth factor-1 (IGF-1) are involved into the regulation of oxidative stress and cell injury (97). Therefore, MMPs (MMP-2, MMP-6) transferred by endothelial cell-derived EVs translates the angiogenic impact of endothelial cells and promotes vascular integrity through VEGF/Erk 1/2 signaling pathway (98). It has been suggested that endothelial cell-derived EVs that are released in response to IL-3 stimulation contain angiopoetic factors, such as micro-RNA-124,−126-3p. Additionally, there are indirect angiopoetic effects that relate to post-ischemic formation of capillary-like structures and collateral vessel formation as a result in delta-like 4/Notch signaling, as well as from the cooperation of EVs with β1 integrin leading to Ras-related C3 botulinum toxin substrate 1-extracellular signal-related kinase 1 and 2-avian erythroblastosis virus E26 homolog-1 signaling and secretion of the CCL2 (99). Moreover, the activation of plasminogen into plasmin at the surface of endothelial cell-derived EVs mediates angiogenic properties of endothelial progenitor cells (100). Finally, support of endothelial structure integrity by EV cargo materials leads to improved endothelial function and a reduction of fibrosis in vasculature and myocardium (101). Previous studies have demonstrated that endothelial cell-derived EVs may promote vascular mineralization after the release of various specific mineralization-promoting cargos (tissue non-specific alkaline phosphatase, annexin-II and annexin-VI) (102, 103). Interestingly, it has identified a specific trafficking protein called sortilin, which was an initial trigger to shape EVs from progenitor endothelial cells, vascular smooth muscle cells, and mononuclears (103). In fact, the secretion of calcifying EVs is under the control of pro-inflammatory cytokines and is probably regulated epigenetically (104). However, the hypothesis regarding that the endothelial cell-derived EVs are embedded onto epigenetic regulation of endogenous repair system mediating tissue protective effects requires further investigation to be clearly understood.

## EV-Derived Non-Coding RNAs in Cardiac and Vascular Remodeling in Heart Failure

There are four epigenetic mechanisms: histone acetylation, histone methylation, DNA hyper- and hypo-methylation, and non-coding RNA regulation. Multiple pre-clinical and clinical studies have shown that non-coding RNAs transferred by EVs are the most important epigenetic regulators of cell differentiation, proliferation, survival, development, regeneration, and neovascularization (52, 105, 106). Interestingly, some subsets of free cell non-coding RNAs, such as mi-RNAs, are normally derived to the target cells by high-density lipoproteins (107), however, the majority of long non-coding RNAs and short chains of mi-RNAs are enriched and stable in EVs and can be delivered by EVs acting as gene regulators (108). Several characteristics of various progenitors cells, which are embedded onto cardiac and vascular remodeling and are expected to carry benefits to the failing heart and vasculature, such as trans-differentiation, paracrine output, migration, survival, are able to be potentially regulated by non-coding RNAs disembarked from endothelial cell-derived EVs (90, 109). For instance, endothelial cell-derived EVs through a transfer of long noncoding RNA Mhrt have exhibited the ability to cause acetylation of myocardin, which plays a pivotal role in re-programming cardiac myocytes (50, 51). There is strong evidence that micro-RNA-1 and mi-RNA−155 via interaction with free fatty acids cardiac binding protein FABP3, insulin-like growth factor-1 (IGF-1), IGF-1 receptor, and twinfilin-1 regulate cardiac myocyte free fatty acids uptake, provide proliferative response, and mediate myocardial hypertrophy (62, 63). Moreover, the spectrum of mi-RNAs that cooperate with impaired insulin sensitivity, insulin signaling, ATP production, ketone bodies, free fatty acids, and amino acids utilization, and thereby impact on cardiac relaxation, contractile function and remodeling, is wide. For instance, mi-RNA-26a,−103, -and 107 have been shown to predominantly be regulators for insulin receptor function and free fatty acid metabolism (53, 110–116). Additionally, recent pre-clinical studies have revealed that mi-RNA-378 and mi-RNA- 451 may play a crucial role in energy metabolism control through interacting with carnitine O-acetyltransferase, the peroxisome proliferator-activated receptor γ coactivator 1β, and LKB1/AMPK-signaling (117–119). However, there is no strong evidence showing that the endothelial cell-derived EVs were cargo for these molecules and this area remains largely unexplored.

Ischemia/hypoxia are triggers for endothelial cells to derive EVs in which were found 66 up-regulated microRNAs for VEGF/Akt and Eph/Ephrin signaling, as well as NO-depending pathway and 119 down-regulated microRNAs for TGF-beta receptor complex and endogenous sterols' synthesis (90, 110). It has been noted that TGF-beta receptor complex pathway, SMAD, and endogenous sterols' synthesis play crucial roles in initiating reperfusion-induced pathological events and fibrotic response (111). Additionally, EVs accumulate in the ischemic myocardium and regulate local inflammatory responses and vascular function through Erk1/2 STAT, Akt/MAPK- NF-κB signaling pathway (69, 112). Therefore, NLRP3 inflammasomes and endothelial cell-derived EVs act as cargo for a wide spectrum of active molecules, including inflammatory cytokines (TNF-α, IL-6, IL-10, IL-18, CRP, HIF-1-α), regulatory peptides (NF-κB), mi-RNAs (-125a,−125,−138,−146,−155a) act IL-1β and IL-18 pathway (69).

There is evidence showing that mi-RNA-21, after a delivery into cardiac myocytes and endothelial cells, have reduced apoptosis through decreases in Programmed Cell Death gene-4 expression, inhibition of the extracellular inhibitor of the sprout regulated kinase 1 (Spry1), and stimulation of the expression of VEGF (53, 67, 113). In animal models, the protective effect of microRNA-21 against ischemia-induced myocardial damage was confirmed by diminished cell apoptosis around the infarcted areas after treatment with antibody vs. miRNA-21 (114). Therefore, mi-RNA-29 has interacted with the genes encoding the extracellular matrix components, such as collagen, fibrillin, and elastin, and thereby reduces the risk of early rupture of the cardiac wall after myocardial infarction (68). In fact, several mi-RNAs were found to be involved in the provision of the myocardial fibrosis and vascular elastosis through interplay with IGF-1/IGF-1 receptor and PI3K/Akt/MAPK- NF-κB signaling pathways that lead to disproportionate distribution and exaggerated arrangement of type I collagen fibers in the extracellular matrix (66). Mi-RNA-378 also had a critical role in the regulation of cardiac fibrosis and the effects of biomechanical stress on cardiac remodeling (120–123). It has been reported that mi-RNA-378 inhibited cardiac fibrosis in EVs-dependent secretory manner, partially via its role as regulator of p38 MAP kinase phosphorylation by targeting MKK6 in cardiac fibroblasts (120).

Interestingly, there are some mi-RNAs (-146a,−155) that were associated with various metabolic comorbidities (type 2 diabetes mellitus, abdominal obesity, resistance to insulin) among patients with HF and adverse cardiac remodeling (70, 108), but the role of endothelial cell-derived EVs in transportation of these molecules still needs to be confirmed further. In contrast, micro-RNA-126 being a component of endothelial cell-derived EVs mediates protein kinase G activity, VCAM-1 expression on the surface of endothelial cells, and increases monocyte recruitment and differentiation (53, 90, 109–119). Several specific mi-RNAs (-92a,−126, and−133) were determined as regulators of microvascular coronary endothelial function and blood coagulation (120, 121), and mi-RNA-138 and−155 were negatively associated with NO production and cell-cell communication, respectively (122, 123). Animal study has revealed that mi-RNA-17-3p-dependent inhibition of TIMP3 can increase cardiac proliferation and endothelial cell survival (124–131). Additionally, mi-RNA-124 and mi-RNA−126-3p were determined to be key epigenetic regulators of PI3K/Akt/MAPK- NF-κB signaling pathways in progenitor endothelial cells, which are a core element of endogenous repair systems (79). The number, activity, and survival of progenitor endothelial cells were found to be significantly reduced in HF and corresponded to poor clinical outcomes (132); consequently, the role of several epigenetic regulators could be investigated in the direction of creating new biomarker predictive models.

There are data that confirm the idea regarding the ability of endothelial cell-derived EVs to be a driver for hypercoagulable phenotypes at the acute phase of decompensated HF in contrast with the well-known platelet-dependent pro-thrombotic state that occurs in HF (133). Probably, endothelial cell-derived EVs may ensure a control for neutrophil extracellular trap formation and pro-thrombotic profile (protein C, thrombin generation, tissue factor supply). However, the impact of these findings on the clinical outcomes among patients with different HF phenotypes and with/without sinus rhythm is not fully understood.

## EVs-Derived Micro-RNAs as Predictive Biomarkers in HF

It has been suggested that exosomal micro-RNAs can be used as predictive biomarkers among HF patients (134). There is evidence that circulating levels of exosomal mi-RNAs (92b-5p,−192-5p, and−320a) in acute decompensated HF patients were significantly higher than in healthy volunteers and that the levels of exosomal mi-RNAs correlated positively with age and cardiac cavities enlargement, and inversely with LVEF and LV fraction shortening. Interestingly, the signature of circulating cell-free mi-RNAs (-423-5p,−320,−22, and−92b) was previously determined as a predictor of HF in patients after dilated cardiomyopathy and myocardial infarction (135–137). Additionally, mi-RNA-126 and mi-RNA-199a, which were contained in EVs, were related to cardiovascular clinical outcomes, whereas the levels of circulating free-RNAs were not associated with HF-related events (138). However, there are several controversies between the data received from different investigators in this issue. For instance, there was no significant difference between HFrEF patients and healthy volunteers in the expression of circulating mi-RNAs between EVs and unfractionated serum (139). In contrast, mi-RNA-192-5p expression was significantly elevated in patients who developed HFpEF within 1 year after acute myocardial infarction compared with healthy volunteers (140). Thus, the discriminative ability of exosomal micro-RNAs remains uncertain and requires further evaluation. Finally, it is not clear whether different phenotypes of HF (HFrEF and HFpEF) are the result of altered cardiac and vascular repair due to certain epigenetic responses, which are yielded by co-morbidities, such as type 2 diabetes mellitus and abdominal obesity (141). In this context, the role of endothelial cell-derived EVs that transfer several biological active molecules, including non-coding RNAs, is not fully understood and should be studied further.

## Future Directions and Challenges in EV Research

The transcriptomics of EVs, including signature of EV-derived microRNAs and RNA-derived fragments, is disputed as a promising source of biomarkers in liquid biopsies (142). Future studies that could clearly explain the potency of EVs as biomarkers for personalized care of HF are required. Probably brand new technological solutions, such as an integrated microfluidic exosome analysis platform, will become powerful non-invasive diagnostic tools for easy screening and monitoring of the EV-based Liquid biopsy (143). There are expectations that EVs will be a promising tool for transfer of the drugs and vector signals to the target cells to regulate many processes involved in myocardium and vasculature reparation, endothelial homoeostasis, and adaptations to myocardial injury (144). These advances have made EV-based point-of-care applications possible and promising, while new devices for use in liquid biopsy need to be developed in the future.

## Conclusion

Endothelial cell-derived EVs have been identified as enveloped particles that are very heterogeneous in size, composition, and biogenesis that play a pivotal role in the evolution of HF, including cardiac and vascular remodeling. Several co-morbidities, such as type 2 diabetes mellitus, insulin resistance, and abdominal obesity, have been found to be closely related to the deterioration of repairs, and an increase in ischemia, inflammation, fibrosis, cardiac hypertrophy, accelerate atherosclerosis, and thereby to mediate shaping of HFpEF or HFrEF. EVs produced by progenitor and mature endothelial cells are co-regulators of these responses influencing HF nature evolution and probably having predictive potency to clinical outcomes. Large pre-clinical and clinical studies are needed to further understand the role of endothelial cell-derived EVs in the pathogenesis of HFrEF/HFpEF and prediction of HF-related events.

## Author Contributions

All authors listed have made a substantial, direct and intellectual contribution to the work, and approved it for publication.

### Conflict of Interest

The authors declare that the research was conducted in the absence of any commercial or financial relationships that could be construed as a potential conflict of interest.

## Abbreviations

ABs: apoptotic bodies
CCL2: chemokine ligand−2
CV: cardiovascular
ECM: extracellular matrix
ERK1/2: extracellular signal-related kinase 1 and 2
EVs: extracellular vesicles
FAP-α: Fibroblast activation protein α
HF: heart failure
HFpEF: HF with preserved ejection fraction
HFrEF: HF with reduced ejection fraction
HIF-1-α: hypoxia-inducible factor-1 α
HSP: heat shock proteins
GDF-11: growth differentiation factor 11
IGF-1: insulin-like growth factor-1
IL: interleukin
LV: left ventricular
MAPK: mitogen-activated protein kinase
Mhrt: myosin heavy chain-associated RNA transcript
miRNA: micro ribonucleic acid
MMP: matrix metalloproteinase
MVBs: multi vesicular bodies
mTOR: a serine/threonine protein kinase
NADF: nicotinamide dinucleotide phosphate
NF-kB: nuclear factor-κB
PS: phosphatidylserine
Rac1: Ras-related C3 botulinum toxin substrate 1
ROCK-2: Rho-associated coiled-coil containing kinase-2
ROS: reactive oxide species
SOD: super oxide dismutase
Spry1: sprout regulated kinase 1
TGF-β: transforming growth factor β
TNF: tumor necrosis factor
VEGF: vascular endothelial growth factor.
